# Vinculin Binding Angle in Podosomes Revealed by High Resolution Microscopy

**DOI:** 10.1371/journal.pone.0088251

**Published:** 2014-02-11

**Authors:** Marie Walde, James Monypenny, Rainer Heintzmann, Gareth E. Jones, Susan Cox

**Affiliations:** 1 Randall Division of Cell and Molecular Biophysics, King’s College London, London, United Kingdom; 2 Institute of Physical Chemistry, Abbe Center of Photonics, Friedrich-Schiller-Universität Jena, Jena, Germany; 3 Leibniz Institute of Photonic Technology, Jena, Germany; Northwestern University Feinberg School of Medicine, United States of America

## Abstract

Podosomes are highly dynamic actin-rich adhesive structures formed predominantly by cells of the monocytic lineage, which degrade the extracellular matrix. They consist of a core of F-actin and actin-regulating proteins, surrounded by a ring of adhesion-associated proteins such as vinculin. We have characterised the structure of podosomes in macrophages, particularly the structure of the ring, using three super-resolution fluorescence microscopy techniques: stimulated emission depletion microscopy, structured illumination microscopy and localisation microscopy. Rather than being round, as previously assumed, we found the vinculin ring to be created from relatively straight strands of vinculin, resulting in a distinctly polygonal shape. The strands bind preferentially at angles between 116° and 135°. Furthermore, adjacent vinculin strands are observed nucleating at the corners of the podosomes, suggesting a mechanism for podosome growth.

## Introduction

Podosomes and the related invadopodia are receiving increasing attention due to their potential involvement in physiological events, such as monocyte extravasation and tissue transmigration, and pathological conditions such as atherosclerosis [1], osteoporosis [2] and cancer metastasis [3]. Normal podosome formation and dissociation accompanies the migration of myeloid cells. However, failure to make podosomes, as in the immunodeficiency disorder Wiscott-Aldrich syndrome, severely compromises the migration and chemotaxis of macrophages and dendritic cells. Podosomes share many similarities with focal adhesions, but in contrast to these more ubiquitous adhesion structures contain proteins that regulate actin polymerization (the Wiskott-Aldrich syndrome protein WASp amongst others [4]), and seem to have the ability to tubulate and remodel the plasma membrane and degrade components of the extracellular matrix [5].

**Figure 1:**
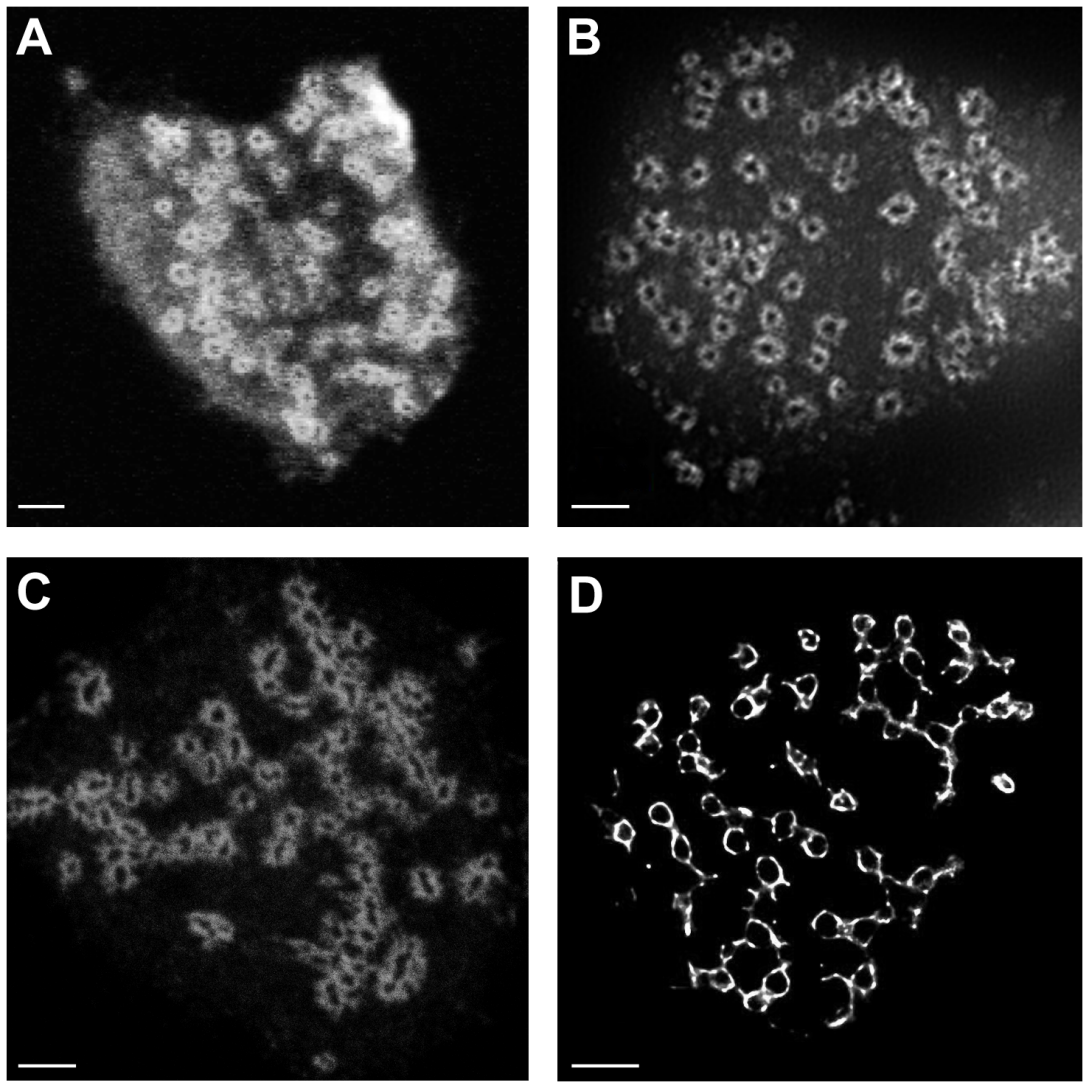
The podosome ring of actin associated proteins in THP-1 cells imaged with four different microscopy techniques. (**A**) A confocal image where vinculin is labelled with Alexa488. (**B**) A SIM image where vinculin is labelled with Alexa488. (**C**) A STED image where vinculin is labelled with ATTO-647. (**D**) A 3B image where vinculin is labelled with Alexa488. All scale bars are 2 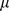
m.

Podosomes are typically located at the ventral cell surface and mark sites of contact with the extracellular matrix or a substratum [6]. Each podosome consists of a core of actin filaments surrounded by a ring containing integrin-associated proteins such as talin, vinculin and paxillin, amongst others [6], [7]. The ring complex connects cell surface integrin with the cytoskeleton. They have a dot or rosette-like appearance and measure 0.5–1.0 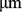 in diameter and 0.2–0.4 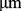 in depth [8]. The integrin-associated proteins, such as vinculin, appear to form round ring-like structures surrounding the acting core when imaged using confocal fluorescence microscopy (Figure 1A). Since their size is close to the diffraction limit (about 200 nm for standard labels) they are prime candidates for super-resolution imaging, as few details of the structure can be seen by standard fluorescence imaging.

The aim of this work was to investigate the structure and composition of podosomes by means of multi-colour high-resolution fluorescence microscopy and investigate whether new details of these structures could be revealed.

We have identified the surrounding ring by labelling paxillin, talin and vinculin, which are a key components of this adhesion structure. Talin is an actin-binding protein composed of a head domain, which can dock onto the plasma membrane and bind actin and integrin, and a rod domain with several vinculin-binding sites. Talin is auto-inhibited in the cytosol, but it has been shown that mechanical stretching forces expose the vinculin-binding sites and thereby allow for the translation of a mechanical signal into a chemical one [9]. Talin is regarded as a structural platform necessary for linking sites of adhesion with the contractile cytoskeleton [10]. Vinculin can bind to talin, paxillin, 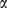-actinin and actin filaments, depending on its structural state. Its head domain regulates integrin dynamics, while the tail domain is involved in the mechanotransduction of forces [11]. Recruitment of vinculin to the podosome ring has been shown to be driven by myosin-dependent tension on the actin network [7]. Paxillin is a multi-domain scaffolding protein that contributes to the coordination of cell adhesion dynamics by interacting with numerous regulatory and structural proteins [12], [13].

One way to achieve a higher resolution than that achieved by standard fluorescence microscopy is to use particles with a shorter wavelength; in particular, resolution at the nanometre scale can be achieved in biological samples using electron microscopy. The actin cores of podosomes have been imaged using scanning electron microscopy (SEM) [14]. A dense actin core is clearly visible, with radiating actin fibres. However, sample preparation for this technique requires removing the plasma membrane, leading to an unquantified level of structural damage to the podosomes. Optical microscopy does not require this damaging sample preparation, and therefore should provide a more reliable structural characterisation; additionally, protein-specific staining is simple to perform for optical microscopy, allowing the structures formed by a number of proteins to be characterised.

Historically, the resolution of light microscopy has been believed to be fundamentally limited by diffraction. However, a number of far-field fluorescent microscopy techniques have recently emerged which overcome this limit [15], either by projecting a pattern onto a wide-field image and extracting and reassembling the down-shifted high frequency information [16], [17], narrowing the effective point spread function (PSF) of a confocal system [18], or localising many blinking fluorophores in a time series [19], [20] and assembling a result image in a pointillistic manner [21]. Each method has limitations on acquisition speed, ease of use, and achievable resolution, and these determine which is the optimal method for a given experiment.

In structured illumination microscopy (SIM) a grating pattern is projected onto the sample, down-shifting otherwise unobservable high frequency information by the Moiré effect so that it is transmitted through the optical system of the microscope. An algorithm extracts the high frequency information from multiple phase shifted raw images, then assembles a high resolution image in real space. In order to achieve isotropic resolution improvement for each plane, at least three different grating orientations with five phase steps for each are required, plus one dark image (total: 16 images). Linear SIM can achieve approximately twice the resolution of conventional wide-field microscopy in all three spatial dimensions [22]. However, reconstructed SIM images can have artefacts (as with all methods involving deconvolution).

Stimulated emission depletion microscopy (STED) is a spot scanning technique which narrows the effective point spread function (PSF) of the microscope. Stimulated emission occurs when an excited fluorophore is hit by light usually at the red edge of its emission spectrum, inducing it to emit a photon of the same red edge wavelength. Photons from molecules remaining in the excited state are stochastically emitted via spontaneous fluorescence. They can thus be spectrally discriminated from photons emitted via stimulated emission, allowing the image to be formed only from remaining fluorescent light. In STED microscopy the sample is scanned with a beam at the excitation wavelength, and simultaneously with a doughnut-shaped beam (with zero intensity at its centre) at the stimulated emission wavelength. Collecting only the non-stimulated light (emitted from a position close to the intensity zero of the doughnut beam) leads to an effectively narrowed PSF. Finally, driving the STED beam towards saturation allows for very high resolution images to be obtained. Resolutions of approximately 30 nm in the lateral (XY) direction and 45 nm in axial (Z) direction have been demonstrated in biological samples [23], [24]. STED images have a considerable advantage that they do not require any postprocessing, and are so easy to interpret, but the resolution can be limited by aberrations produced by the sample.

Localization-based methods such as STORM [19] and photo-activation localization microscopy (PALM [25], fPALM [20]) exploit the fact that a point source can be localised to far greater accuracy than the resolution of the system. A high-resolution image (approx. 20 nm in XY) is reconstructed from individual molecule positions [19]. Imaging techniques that use astigmatism [26] or double-plane detection [27] have also achieved Z-resolution well below 100 nm. However, the acquisition time scale is generally minutes, making STORM poorly suited to live cell imaging [28]. Bayesian analysis of blinking and bleaching (3B analysis) is an alternative localization-based method which analyses the entire dataset as arising from a number of fluorophores. This allows it to deal with significantly more dense datasets, allowing it to reconstruct superresolution images of 50 nm resolution from data taken over 4 s [29].

Here we demonstrate that commercial SIM and STED systems can be comparable in resolution when imaging cytoskeletal structures on the cell membrane.

## Results

**Figure 2:**
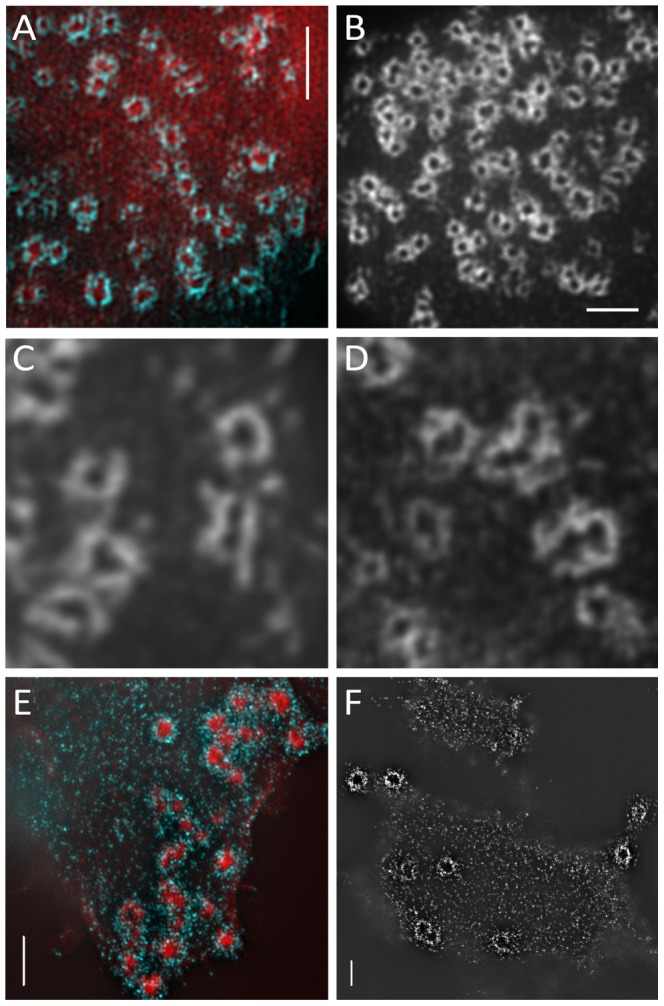
SIM images show polygonal structure of podosomes. (**A**) Immunolocalisation of actin (red) and vinculin (blue) in podosomes. SIM image of podosomes recorded with 3 different grating directions and 5 grating phase positions showing the actin core surrounded by an vinculin-enriched protein ring. (**B**) SIM images of vinculin rings of podosomes, imaged with 5 different grating directions. The podosome rings appear to have a polygonal structure with both 3 and 5 grating directions. (**C, D**) SIM images of vinculin rings. Vinculin strands on the corners of podosomes are visible. (**E, F**) Immunolocalisation of actin (red) and paxillin (blue) in podosomes. SIM images were recorded with 5 grating directions and 5 grating phase positions. Paxillin rings appear polygonal, too, but are more punctate than the vinculin rings. All scale bars are 2 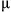
m.

Both SIM and STED were used to image the vinculin ring of podosomes, with the SIM measurements also imaging actin (Figure 2A). Podosomes were found to be widely distributed over the substratum-attached side of the cell. It was clearly visible that each podosome consisted of an actin core surrounded by a vinculin-rich ring, as expected from the current podosome model. However, the vinculin rings appeared to be polygonal rather than round (Figure 2B–D). For SIM, images were taken with both three and five grating directions, with the observed structures remaining the same.

**Figure 3:**
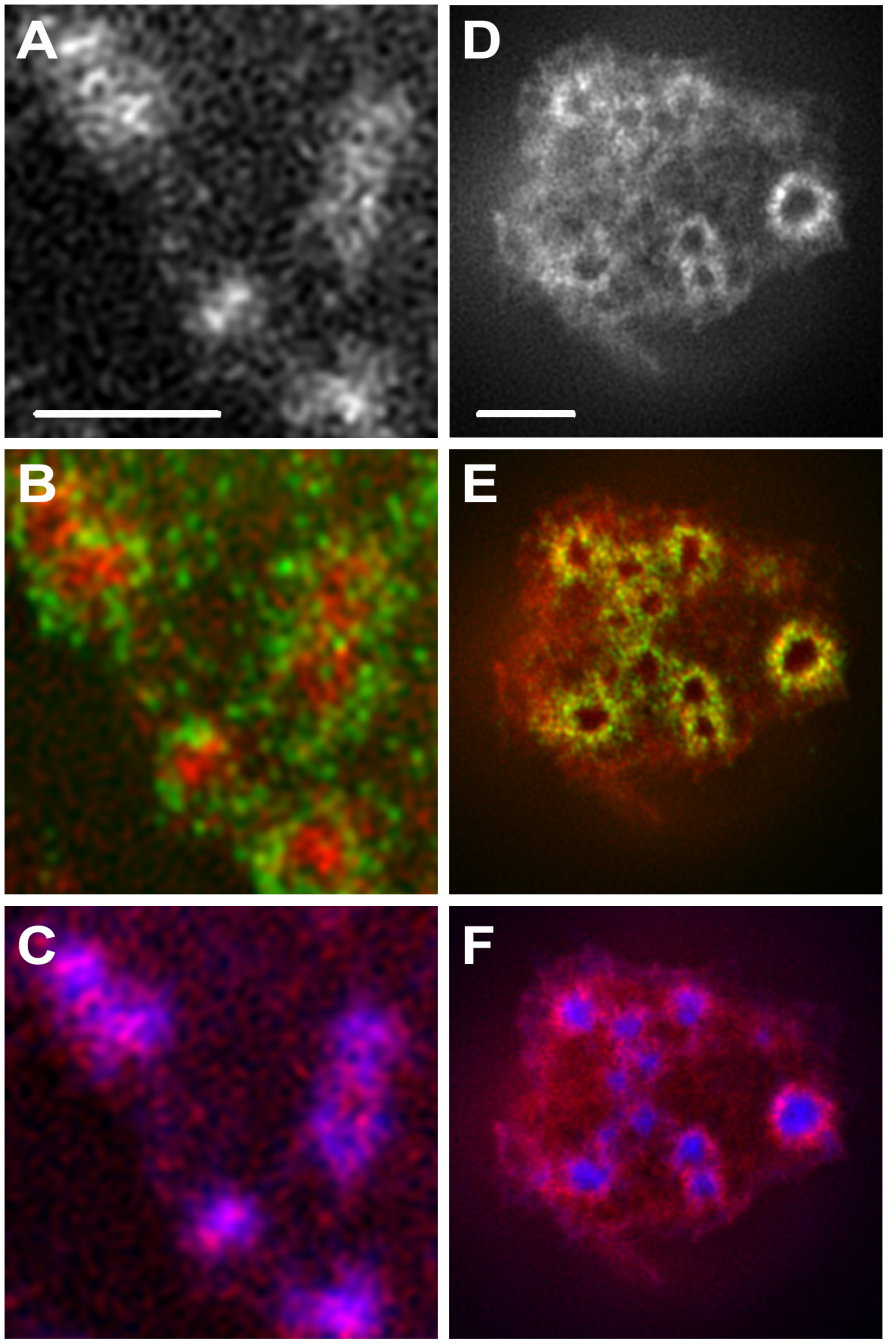
Talin was observed in two different configurations. (**A–C**) Left side: Talin is localized at the podosome core, possibly due to podosome dissociation. (**D–F**) Right side: Talin is localized in the adhesion ring in steady-state podosomes. (**A, D**) SIM immunolocalisation of talin. Scale bars are 1 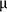
m. (**B, E**) SIM immunolocalisation of talin is shown in red and paxillin (*B*) or vinculin (*E*) in green. (**C, F**) SIM immunolocalisation of talin is shown in red and actin in blue.

Samples stained for paxillin and F-actin showed similar polygonal structures for paxillin to those seen for vinculin. These structures did not appear to be made up of straight strands, as observed for vinculin, but much more punctate (Figure 2E, F). We noticed two different talin configurations: it can be localized at the podosome core, co-localizing with actin (Fig. 3A–C) or in the adhesion ring (Fig. 3D–F). In many cases, Talin was observed to co-localize with vinculin forming very similar rings (Figure 3E).

**Figure 4:**
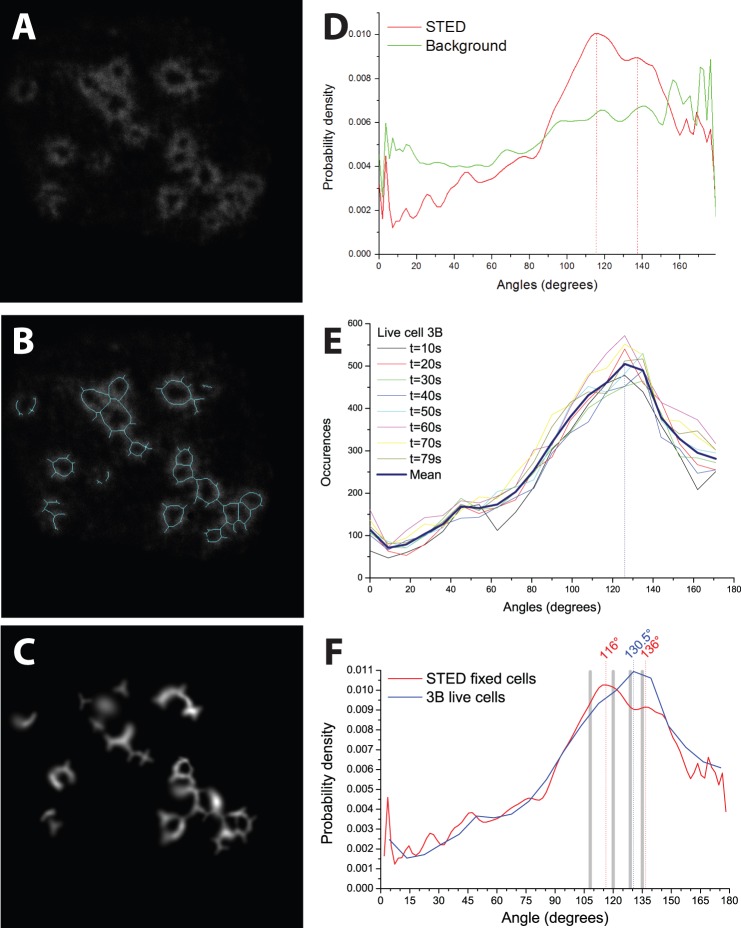
STED images reveal dominant vinculin binding angle in podosomes. (**A**) STED image of vinculin rings of podosomes. The vinculin rings appear distinctly polygonal, with vinculin strands apparently nucleating from corner points. (**B**) Skeletonisation of image *A* shown in blue. (**C**) Result of fitting blurred line segments to the underlying image *A* at corners shown in *B*. The intensity of the Gaussian line segment is displayed. The background and line segments wider than 10 pixels are not displayed for clarity. (**D**) Probability distribution of angles at vinculin corners in STED images of fixed cells: a peak at 116° with a shoulder at 136° is observed above the background. (**E**) Localisation microscopy on live cells shows that the binding angle in podosomes fluctuates in the range of 120° to 135° (mean peak 130.5°
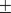
5°), but is not time-dependent. (**F**) Overlay of results from *E* & *F*. Dotted lines indicate dominant binding angles. Grey bars represent the inner angles of flat pentagons (108°), hexagons (120°), heptagons (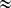
128.6°) and octagons (135°).

The polygonal structures appeared to be made up of straight strands of vinculin 400–600 nm long. Primarily pentagonal and hexagonal shapes were visible (Figure 2C, D). Joining points between two vinculin strands often had enhanced intensity, suggesting a higher concentration of vinculin. Vinculin strands were observed extending from the corner points of the structures, suggesting that new podosome growth is nucleated from the corners. Strands that connect neighbouring podosomes were observed as well (Figures 2C, D and 4B). Since the two dominant types of polygons observed have a similar binding angle, we measured the angles at which the strands of vinculin joined, to see if there was a dominant binding angle for vinculin strands within podosomes.

To achieve this, first the image was skeletonised (reduced to a set of lines) (see Figure 4C–D). Figure 4E shows the fit of the skeletonisation to the original image. The values of all intersection angles were plotted as histograms. Figure 4F shows a probability distribution of the angular distribution for the 1761 corner points over four STED images including Figure 4A–C. These images were selected because they displayed distinct podosome structures in which the vinculin strands were well defined. In other images, podosomes could be partially joined into ill-defined structures, making analysis difficult, or the vinculin strands could appear thicker, probably due to the lack of z-resolution in our STED system (700 nm). The probability distribution is plotted against a control for bias (see Methods section). It can be clearly seen that while the skeletonisation does appear to have an intrinsic trend, there is a clear peak above background when the podosomes are correctly identified. The modal binding angle of vinculin strands in the STED images was found to be 116° with a shoulder at 135° (Figure 4D).

The skeletonisation analysis was also performed on the SIM images. However, the background distribution for the SIM images was found to have an inherent bias towards values just above 100°, probably because the deconvolution process leads to artefacts in the background which intersect at angles determined by the number of grating directions used.

We also carried out live cell imaging with 3B localisation analysis to find out more about the dynamics of the binding angles. Images were taken every 10 s (9 s for the last image). Analysis of these images showed that the binding angle in podosomes fluctuates with peaks between 120° and 135° with mean peak at 130.5°±5° (Fig. 4E). For comparison, the inner corners of flat, regularly shaped pentagons (108°), hexagons (120°), heptagons (128.6°) and octagons (135°) are given as grey lines (Fig. 4F).

**Figure 5:**
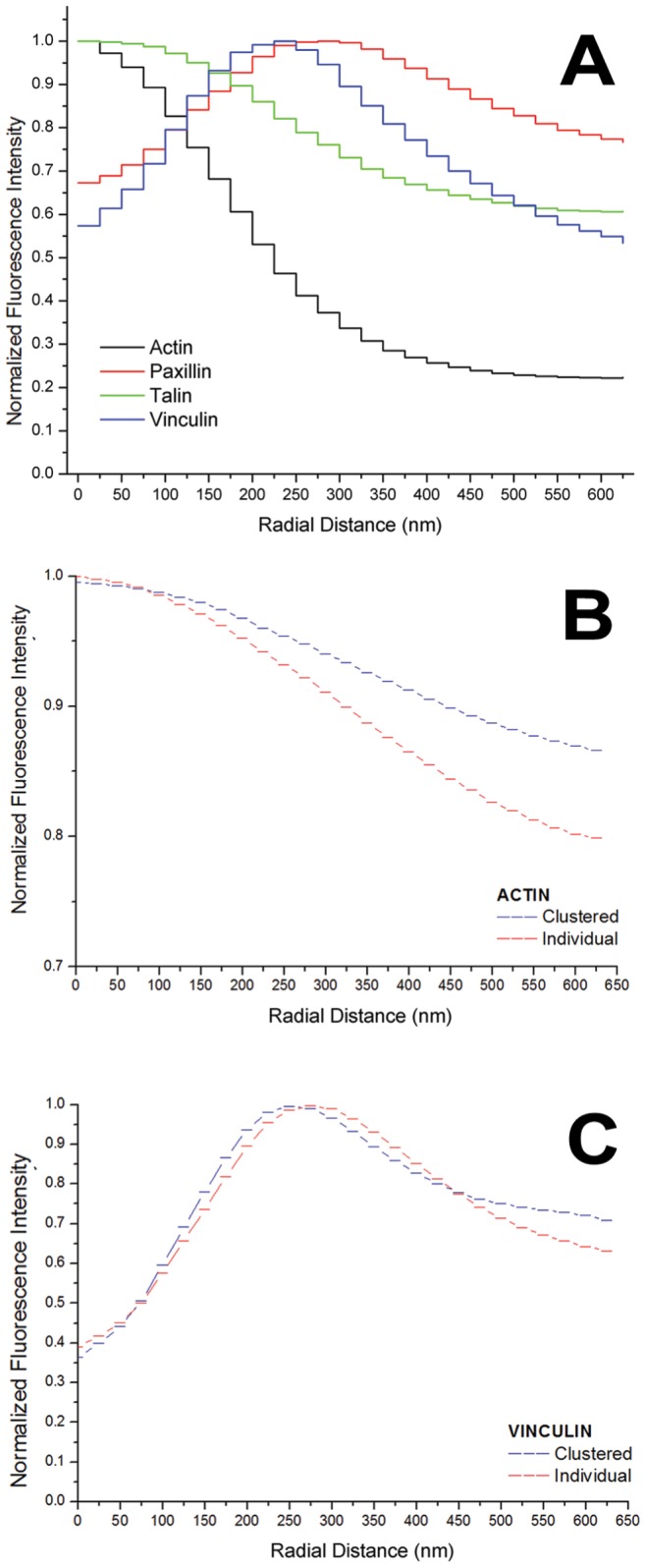
Immunolocalisation analysis and comparison of clustered and individual podosomes. (**A**) Radial distributions of actin (black), paxillin (red), talin (green) and vinculin (blue). The normalized average intensity of the immunolocalisation is plotted with respect to the radial distance from the centre of the actin core. (**B+C**) Comparison of actin (*B*) and vinculin (*C*) distributions in clustered (blue) and individual cells (red).

In order to quantify the immunolocalisation of the ring proteins with respect to the actin core, podosomes were automatically identified in Matlab with a recently published algorithm [30], which separates podosomes in samples stained with phalloidin (for F-actin) based on intensity, size and shape. This algorithm has previously been applied to wide-field and confocal images of human dendritic cells [30]. After segmentation, the distribution of ring proteins can be measured. Here, we calculated the average normalized intensity in a podosome with respect to the distance in nanometres from the centre of its actin core. Ring masks of 25 nm thickness and increasing radius around the core were analysed. Protein distributions around the actin cores were evaluated in 480 podosomes stained for actin, 339 for paxillin, 401 for talin and 141 for vinculin. The resulting normalized intensity distributions are shown in Figure 5 and results are summarized in Table 1. Paxillin and vinculin form distinct rings with peaks at distances *d_pax_*≈275 nm and *d_vin_*≈225 nm. The paxillin ring is broader (FHWM*_pax_*≈385 nm) than the vinculin ring (FHWM*_vin_*≈320 nm). In the histogram, talin appeared almost homogeneously distributed, because the calculated average distribution contains a mixture of the different observed configurations and possibly intermediate situations.

**Table 1:** Results of immunolocalisation distance analysis.

Protein	Plot colour	Peak of distribution	FWHM	*R* ^2^ of Gaussian fit
		[nm] from core centre	[nm]	
Actin	black	0 [ref]	349	0.9888
Paxillin	red	275	385	0.9025
Talin	green	0	244	0.9904
Vinculin	blue	225	320	0.9709

Radial distance of the peaks, FWHM and *R* ^2^ of fit to Gaussian is given for fluorescence intensity distributions of different proteins of the podosome scaffold. Distributions are plotted in Fig. 5.

Furthermore we investigated whether the distributions of actin and vinculin in podosomes that occur in clusters differed from individual ones. A distance matrix for all identified podosomes was calculated and a threshold 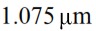 of mean distance to the 3 closest neighbours was determined to select clusters. 207 clustered and 297 individual podosomes were analysed. The resulting average normalized intensities with respect to the distance from the core are plotted in Fig. 5A&B. The typical distance (core-to-core) between neighbouring clustered podosomes was 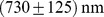, while actin cores of individual podosomes were on average 
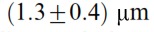
 apart. Differences concerning the vinculin binding angle between clustered and peripheral podosomes were not found. Actin cores did not appear polygonal in our images, possibly because of limited optical resolution.

## Discussion

Two-colour SIM and STED imaging reveals previously unnoticed detail of podosome architecture. Immunofluorescent staining of paxillin and vinculin shows rings surrounding the actin core, confirming the current podosome model. However, their shape does not appear round, but exhibits distinct corners. This is unexpected since the actin structures observed using SEM do not appear polygonal [14], and when using optical imaging methods at standard diffraction-limited resolution the vinculin ring appeared roughly circular. The geometrical structure of vinculin is in agreement with with 3B localization microscopy results [29].

Different talin localizations were observed. Talin localised in the centre of the podosome suggests podosome dissociation in which talin is drawn to the centre and eventually removed, as revealed by live cell microscopy [29]. At steady-state, the talin ring co-localizes roughly with vinculin.

With a skeletonisation algorithm we identified a modal binding angle of around 116° in the vinculin rings of podosomes in our STED data. To control for bias in the skeletonisation algorithm a chi-squared test was used. A distinct modal binding angle is already known for filaments such as those of the actin core which bind at 70° [31]. This dominant binding angle is thought to arise from interaction between actin and the nucleator known as the Arp2/3 complex [32]. We note that the binding angle we observe for vinculin is close to the outer binding angle of actin (180°–70° = 110°), and that actin strands have been observed in the region of the vinculin rings [33]. 3B localisation microscopy of live cells showed that the binding angle in live podosomes fluctuates on a time scale of seconds in the range of 120° to 135°.

Image analysis of our immunolocalisation experiments shows that the different proteins of the ring have different localizations within the podosomes as suggested in previous studies [29], [30]. We confirm that the vinculin ring is closer to the core than the paxillin ring. The diameter (FWHM) of the actin core was found to be approximately 350 nm, which matches other fluorescence and EM studies.

The morphology of clustered and individual podosomes was found to be very similar. While the typical distance between vinculin ring and podosome centre remained the same, actin levels were higher in the periphery of clustered podosomes. Actin fibres are known to radiate from podosome cores. A likely explanation for the increased actin levels are interactions between neighbouring podosomes [14].

Vinculin strands that are observed at the corners of podosomes may be starting points for the self-assembly of new podosomes. This observation strongly suggests that new podosomes can nucleate from the corners of existing structures. In another study using 3B localization microscopy we were able to show evidence for this hypothesis [29]. We also observed podosomes that appeared to be connected to each other via vinculin strands. It raises a number of interesting questions with regard to how the podosomes form: Does the vinculin structure of podosomes predominantly form by a strand being continuously laid down from one nucleation point? What determines when a new strand in a different direction is initiated? And what is the relationship between the formation of the actin core and the protein-enriched ring?

The fact that the modal vinculin binding angle is in the range of the inner angle in flat pentagons (108°) and hexagons (120°) to heptagons (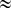
128.6°) and octagons (135°) suggests that podosomes may tile the cell adhesion sites with a mix of polygonal structures. Mixed hexagonal and pentagonal tiling is frequently found on curved surfaces (the tiling pattern of a football being a nice example). The choice between different lattices allows for adaptation to arbitrary surface shapes, which is probably relevant for migrating podosome-forming cells *in vivo*. In such a lattice, vinculin has a high packing efficiency: the attachment area can be covered with minimal amounts of vinculin molecules.

We have demonstrated that high resolution fluorescence microscopy makes the observation of the fine structure of biological samples below the diffraction limit possible, allowing us to see previously unnoticed details of the protein architecture of podosomes. In combination with image processing techniques, a mathematical analysis of these features allows for the characterization of the geometrical attributes of sub-cellular structures.

## Materials and Methods

### Microscopy Setups

The confocal system used for Figure 1 was a Zeiss LSM 510 confocal using a 63×objective. The SIM system is a commercial prototype based on the Elyra S by Carl Zeiss Jena (Germany). Available excitation light sources were 405 nm, 489 nm, 561 nm and 635 nm laser lines. For structured illumination, three different gratings were used, one 29 lines/mm grating and two 41.2 lines/mm gratings. The grating position and axial position of the sample table were controlled by piezo controllers (Physik Instrumente, Germany). Images were recorded with a CCD camera (Andor, USA), cooled to −50°C. Reconstructions were done with the commercial ZEN software installed on the system (based on [34]). The STED system is the commercial TCS-SP5 STED system from Leica (Germany). Excitation was carried out by a 635 nm pulsed diode laser and de-excitation by a MaiTai tunable fs laser at 750 nm. A resolution of 80 nm has been demonstrated with this system using bead samples.

### Cell Culture & Immunocytochemistry

All samples were prepared from the THP-1 human monocytic leukaemia cell line, which was obtained from the ATCC collection (LGC Standards, Middlesex, UK). This cell line was stimulated to differentiate into macrophages. Podosome formation was induced in these cells by seeding them on fibronectin coated cover glasses in the presence of the cytokine TGF-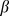-1 (1 ng/ml). Vinculin-staining was conducted using VN-1 antivinculin mouse monoclonal antibody (Sigma, UK), conjugated to an anti-mouse secondary antibody. For Paxillin-staining anti-paxillin mouse monoclonal antibody (Sigma, UK) conjugated to an anti-mouse secondary antibody was used. The conjugates for SIM and confocal samples were Alexa488 and Alexa568 and for STED samples ATTO anti-mouse 647N (Sigma). For parallel labelling of vinculin and paxillin, a vinculin-GFP-lifeact construct was expressed. Actin was visualized by staining samples with Alexa488-, Alexa568- or Alexa633-conjugated phalloidin (Molecular probes). For talin experiments, THP-1 cells expressing an mCherry-tagged truncated talin construct were used (for transduction details see [29]).

### Image Processing

In order to measure the angles at which the strands of vinculin joined, the images were skeletonised (reduced to a set of lines). The resulting lines will be 8-connected, where a pixel can be connected to any of its eight nearest neighbours. This was used to identify corners where vinculin strands joined, and then the raw image in the region of these corners was fitted by modelling the corner as a number of line segments.

Skeletonisation reduces an image to a set of lines which largely preserve the connectivity of the image [35]. First the images were thresholded, then repeated morphological thinning was carried out with the hit-and-miss transform, which matches pixel shapes with a specific pattern. Eight kernels, all rotations of a single kernel, were used. The resulting images were pruned by finding all endpoints and junctions, and removing all endpoint-junction segments that were less than 150 nm long (see Figure 4D, which shows the skeletonisation of Figure 4C). Next, all “corners” of this skeleton were identified: Corners were defined as all such points where 3 or more lines join. These types of corners were present in those STED images where vinculin strands could be observed at the corners of podosomes. We then fitted a model which consists of several blurred line segments radiating from a point. The position of this point is the position of the corner, and the number of line segments is determined by the number of lines at the corner. The line segments are blurred with a Gaussian. The model is then fitted to the underlying image, keeping the position of the corner fixed and allowing the blur, segment intensity and intersection angle to vary.

The probability distribution was obtained using kernel density estimation [36]. The control for bias in the skeletonisation process was performed by changing the intensity threshold to an extremely low value so that the skeletonisation result obtained did not relate to the podosome structure. The observed angular distribution was compared using a chi-squared test to two null hypotheses: a uniform background and the background observed with incorrect skeletonisation. Both tests found 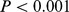, confirming the significance of the result.

The core distance analysis is based on the automated podosome detection by Meddens et al [30] and was written in Matlab (The Mathworks, US) using the DIPimage toolbox (TU Delft, NL).
